# Formulation, Optimization and Evaluation of Luteolin-Loaded Topical Nanoparticulate Delivery System for the Skin Cancer

**DOI:** 10.3390/pharmaceutics13111749

**Published:** 2021-10-20

**Authors:** Imran Kazmi, Fahad A. Al-Abbasi, Muhammad Shahid Nadeem, Hisham N. Altayb, Sultan Alshehri, Syed Sarim Imam

**Affiliations:** 1 Department of Biochemistry, Faculty of Science, King Abdulaziz University, Jeddah 23443, Saudi Arabia; fabbasi@kau.edu.sa (F.A.A.-A.); mhalim@kau.edu.sa (M.S.N.); hdemmahom@kau.edu.sa (H.N.A.); 2 Department of Pharmaceutics, College of Pharmacy, King Saud University, Riyadh 11451, Saudi Arabia; salshehri1@ksu.edu.sa

**Keywords:** luteolin, vesicles, irritation study, optimization, topical gel

## Abstract

In the present study, luteolin (LT)-loaded nanosized vesicles (LT-NVs) were prepared by a solvent evaporation–hydration method using phospholipid and edge activator. The formulation was optimized using three factors at a three-level Box–Behnken design. The formulated LT-NVs were prepared using the three independent variables phospholipid (A), edge activator (B) and sonication time (C). The effect of used variables was assessed on the vesicle size (*Y*_1_) and encapsulation efficiency (*Y*_2_). The selection of optimum composition (LT-NVopt) was based on the point prediction method of the software. The prepared LT-NVopt showed the particle size of 189.92 ± 3.25 nm with an encapsulation efficiency of 92.43 ± 4.12% with PDI and zeta potential value of 0.32 and −21 mV, respectively. The formulation LT-NVopt was further converted into Carbopol 934 gel (1% *w*/*v*) to enhance skin retention. LT-NVoptG was further characterized for viscosity, spreadability, drug content, drug release, drug permeation and antioxidant, antimicrobial and cytotoxicity assessment. The evaluation result revealed optimum pH, viscosity, spreadability and good drug content. There was enhanced LT release (60.81 ± 2.87%), as well as LT permeation (128.21 ± 3.56 µg/cm^2^/h), which was found in comparison to the pure LT. The antioxidant and antimicrobial study results revealed significantly (*p* ˂ 0.05) better antioxidant potential and antimicrobial activity against the tested organisms. Finally, the samples were evaluated for cytotoxicity assessment using skin cancer cell line and results revealed a significant difference in the viability % at the tested concentration. LT-NVoptG showed a significantly lower IC_50_ value than the pure LT. From the study, it can be concluded that the prepared LT-NVoptG was found to be an alternative to the synthetic drug as well as conventional delivery systems.

## 1. Introduction

Cancer is a major health issue all over the globe caused by abnormal cell growth with invasive potentials [1]. There are multiple influencing factors such as genetic factors, environmental factors, alcohol consumption, smoking, exposure to radiation and heredity. Melanoma is a type of skin cancer with the highest metastatic effect rate. It can spread to the other sites of the body by entering into the lymphatic system and bloodstream [2]. It can originate from the malignant transformation of melanocytes and is the most aggressive skin cancer. It has a low survival rate, high multidrug resistance and common relapse. Nowadays, nanoformulations are the most widely explored delivery systems for skin-related disease. They can bypass the effect of the first pass through the liver, with high stability and low dose, and can target the affected area [3,4].

Nowadays much attention has been given to the bioactive compounds with antioxidant properties in the treatment of cancer. The flavonoid luteolin (LT) is an important natural antioxidant that has potent anticancer effects. It is a natural flavonoid, present in different plant species. It has been reported to have a wide range of pharmacological actions such as anti-inflammatory, anti-allergic, antioxidant and anticancer properties. The anti-inflammatory activity of LT is related to its anticancer properties [5]. There are numerous research studies that reported cell line activity against different cancers [1]. It acts by exhibiting cell cycle arrest during the G1 phase linked to suppression of CDK2 activity [6]. It helps to reduce the epidermal growth factor-induced markers as well as restoration of cell–cell junctions [1,7]. LT-loaded nanoformulations such as folacin-modified nanoparticle [8], nanoparticles [9,10], NLCs [11], folic acid-modified ROS-responsive nanoparticles [12], and nanospheres [13] have been prepared and enhanced bioavailability and efficacy have been reported. However, the application of LT vesicles in the skin cancer has not been reported.

The application of nanoformulations has been found effective in the enhancement of solubility of poorly soluble drugs [14]. Over the last decades, lipid-based nanovesicles for topical delivery have been used to improve therapeutic efficacy. There are different types of nanovesicles such as transferosomes, ethosomes, niosomes and cubosomes which are used as topical delivery [15]. These vesicles are composed of cholesterol, phospholipids, surfactants and water. These carrier systems can encapsulate both hydrophilic and hydrophobic drugs. They can deliver drugs to both topical as well as systemic circulation [16]. The use of an edge activator in the formulation of lipid vesicles gives flexibility to the lipid bilayer and can permeate into a very low skin pore size [17]. It is also termed an ultra-deformable vesicle with an aqueous core surrounded by the lipid bilayer. Due to the ultra-flexibility, it can penetrate into the intact human skin and act as non-invasive targeting. The vesicles have the ability to protect a drug from unfavorable absorption into the cutaneous blood vessels. This helps to retain the drug at the skin site [18,19]. The edge activator promotes skin permeation through an intercellular lipid matrix by mixing with stratum corneum as well as by altering the lamellae [18,20,21]. It can permeate with low-, medium- and high-molecular-weight drugs [22].

The object of the present study was to prepare luteolin-loaded nanovesicles (LT-NVs) and characterize them for different parameters. The present delivery systems were optimized by using three factors, phospholipid 90 G (A), edge activator (B) and sonication time (C), at three levels (−, 0, +). The different formulation compositions were assessed on the particle size (PS) and encapsulation efficiency (EE) to select the optimized formulation. From the formulation design approach, optimized luteolin nanovesicles (LT-NVopt) were selected and characterized for permeation, drug release, antioxidant activity and cytotoxicity activity.

## 2. Material and Methods

### 2.1. Materials

Luteolin was purchased from Beijing Mesochem Technology Co. Pvt. Ltd. (Beijing, China). Phospholipid 90G was received as a gift sample from Lipoid GmbH, Ludwigshafen, Germany. Sodium cholate, methyl paraben, triethanolamine and tween 80 were purchased from Sigma Aldrich, St Louis, MO, USA and Loba Chemie, Mumbai, India. Optimization of the formulation was performed using Design Expert software (Stat-Ease, Minneapolis, MN, USA). The cells were procured from the National Centre for Cell Science, Pune, India. Carbopol 934 and disodium hydrogen phosphate were procured from Loba Chemie Pvt. Ltd. Mumbai, India and Planet Science, Vadodra, India. The solvents methanol, chloroform, ethanol and water were used at analytical grade.

### 2.2. Optimization

The prepared LT-NVs were statistically optimized by a three factors, three level Box–Behnken design. LT-NVs were prepared using the variables phospholipid (A), edge activator (B) and sonication time (C) at three-levels, i.e., low (−), medium (0) and high (+) (Table 1). The design showed fifteen formulation runs with three center points (the same composition to check the error) and their effects were assessed on PS (*Y*_1_) and EE (*Y*_2_). The polynomial equation and 3D and contour plots were generated for all the variables to evaluate the individual as well as combined effects. The actual and predicted values were also generated from the software to confirm the results. The selection of formulation variables was done based on the preliminary study. The dose of LT was fixed in all compositions.

### 2.3. Formulation of Luteolin Nanovesicles (LT-NVs)

LT-NVs were prepared using a solvent evaporation hydration method as per a reported procedure with slight modifications [23]. The ingredients phospholipid 90 G (A) and edge activator (B, sodium cholate—tween 80 blends, 50:50) were taken in specified amount as shown in Table 2. Each ingredient including LT (25 mg) was accurately weighed and transferred to a round bottom flask containing chloroform:methanol (10 mL, 1:1). The flask was attached to a rotary evaporator and the organic solvent was removed at low temperature (40 °C) to form a thin lipid film. The flask was removed from the evaporator and kept overnight to remove the traces of organic solvent. LT-loaded thin lipid film was hydrated with phosphate buffer saline (10 mL, pH 6.8) at 100 rpm for 30 min. The prepared luteolin lipid vesicles (LT-NVs) were kept overnight at room temperature for stabilization of vesicles. Finally, the LT-NVs were probe sonicated in ice condition (4 °C) for different time points with 5 min interval to reduce the size. The prepared samples were transferred to a vial and then stored for further characterization.

### 2.4. Vesicle Characterization

The prepared LT-NVs were evaluated for particle size (PS), size distribution (PDI) and surface charge (ZP). The samples were analyzed by a particle size analyzer (Malvern zeta sizer, Malvern, UK). The samples (0.1 mL) were taken, diluted with double distilled water and scanned for PS and PDI. ZP was also evaluated using a similar method with an electrode-containing cuvette to measure the surface charge. The ideal ZP and PDI value must be ±30 mV and less than 0.7 to get uniform vesicle size distribution [24,25].

### 2.5. Encapsulation Efficiency (EE)

LT encapsulation from the prepared LT-NVs was evaluated by an ultracentrifugation method [26]. The formulations (5 mL) were taken in centrifuge tube and centrifuged at 10,000 rpm for 1 h. The supernatant containing LT was collected and diluted further to evaluate LT content in each sample by using UV spectrophotometer (Shimadzu 1800, Kyoto, Japan). The concentration of LT was calculated by the equation:

(1) $$\mathrm{EE}\;(\%)=(\frac{Wa-Wb}{Wa})\;\times \;100$$

*Wa*: Initial LT content; *Wb*: Free LT content.

### 2.6. Formulation of Luteolin Nanovesicles Based Gel (LT-NVoptG)

The optimized formulation (LT-NVopt) was converted into the semisolid gel formulation using carbopol 934 as gelling agent. The previously optimized gelling agent Carbopol (1%, *w*/*v*) was dispersed into the distilled water and kept aside for 24 h for complete swelling. The prepared LT-NVopt was added to polymer dispersion with continuous stirring to get a uniform homogenous gel. Triethanolamine and methyl paraben was added to maintain the pH and preservation to the gel system [27].

### 2.7. LT-NVoptG Characterization

The prepared LTNVoptG was characterized for different parameters to evaluate the characteristics of the gel. The different parameters such as drug content, pH, viscosity and spreadability were evaluated. The drug content was evaluated to calculate the LT amount in the gel formulation. The difference in the amount of LT added and the amount of LT present was calculated. The prepared LT-NVoptG (50 mg) was taken and dissolved in methanol. The sample was centrifuged at 10,000 rpm for 10 min and the supernatant was collected. The supernatant was further diluted, filtered and the drug content was estimated by UV spectrophotometer. The pH of prepared gel was evaluated using a digital pH meter [28]. The gel sample was taken in a small beaker and the pH meter was dipped into it until it showed a stable value. The viscosity was evaluated to check the flow property of the prepared LT-NVoptG by a viscometer at room temperature [29]. The gel was further evaluated for extrudability and spreadability. The gel sample was taken and kept on the glass slide with a pre-marked area. Then, another slide was placed over the sample and weight was applied. The spread of gel after application of weight was noted and the difference between the initial area and final area was calculated [30]. The extrudability of the gel was evaluated by filling the gel sample into the tube and weight was applied. The tube was pressed from the crimp side end and the extruded gel was collected to calculate the extrudability.

### 2.8. Drug Release

The release study from the prepared LT-NVoptG, LT-NVopt and pure LT were evaluated using a dialysis bag [31]. A 2 mL (5 mg LT) sample was filled in the dialysis bag and both the ends were tied. The bags were dipped into the dissolution medium (500 mL with 1% tween 80, pH 6.8) and temperature was set at 37 ± 0.5 °C with stirring speed of 50 rpm. At specific time intervals, the released LT content (5 mL) was collected and replenished with the same volume to maintain the uniform condition throughout the study. The released content from the samples was evaluated by UV spectrophotometer.

### 2.9. Permeation Study

The comparative permeation study was performed using egg membrane following a reported procedure with slight modifications [32]. Egg membrane has similar properties to stratum corneum of human skin [33,34]. The different samples of pure LT, LT-NVopt and LT-NVoptG were taken and filled to the diffusion cell with effective surface area of 3 cm^2^ and receptor volume of 20 mL. The egg membrane was carefully removed and checked for any damage [35]. The samples (~5 mg LT) were filled in the donor compartment and the receptor compartment was filled with phosphate buffer saline. The study was performed at 37 °C with continuous stirring. After specific time points, the permeated content (1 mL) was collected and replenished with fresh release media. The permeated contents were filtered and diluted further with appropriate solvent. The drug content at each time point was measured using spectrophotometer in triplicate (n = 3).

### 2.10. Antioxidant Assessment

The prepared LT-NVopt, LT-NVoptG and pure LT were evaluated for DPPH-based antioxidant activity. The samples were reacted with ά,ά-diphenyl-β-picrylhydrazyl (DPPH) standard to change the color from violet to colorless [36]. The antioxidant has the property to donate the hydrogen ion and decrease the absorbance of the test compounds. The pure LT and LT-NVoptG were prepared in different concentration ranges and sample volume 100 µL was transferred to small glass vials. The samples were incubated with standard DPPH solution and kept aside for 30 min to complete the reaction mixture. Finally, the sample plate was assessed at 517 nm. The study was performed in triplicate and the effects were calculated using the equation:

(2) $$\mathrm{AA}\;\%=\frac{\left(\mathrm{Absorbance}\;\mathrm{of}\;\mathrm{control}\;-\;\mathrm{Absorbance}\;\mathrm{of}\;\mathrm{test}\right)}{\mathrm{Absorbance}\;\mathrm{of}\;\mathrm{control}\;}\times 100$$

### 2.11. Antimicrobial Activity

The prepared LT-NVoptG was evaluated for antibacterial activity and results were compared with pure LT. The study was performed using the microdilution test with a slightly modified reported method [37]. The samples were tested against the microorganisms *S. Aureus*, *E. coli* and *B. subtilis*. The organism’s broth culture was added to the growth medium and transferred to a clean sterilized Petriplate at 121 °C (15 PSI). The plates were kept aside for the solidification of the media. The wells were prepared with a sterilized stainless-steel borer. Each sample was transferred to the well and plates were kept aside at room temperature to diffuse the sample into the medium. The plates were kept in an incubator and the zone of inhibition was measured to check the effect of sample.

### 2.12. Cytotoxicity Study

The cytotoxicity assessment was evaluated to check the effect of the prepared LT-NVoptG, LT-NVopt and pure LT on the skin cancer cell line (B16F1). The cells were collected and stored in CO_2_ (5%) and oxygen (95%) at 37 °C by using Dulbecco Modified Eagle media with the support of serum of fetal calf (5%). The experiments were performed with asynchronous populations in the phase of exponential and rapid growth, 24 h after the plating of a sample [38]. B16F1 cells (3 × 10^3^) were added to DMEM (200 µL) and placed in the microplate (96 plate). The fresh medium was replaced after 24 h incubation time with serum-free DMEM. The cell line was incubated with pure LT, LT-NVopt and LT-NVoptG in the media corresponding to a concentration between 10–1000 µM for 24 h. Then, MTT was added into the well of the microplate and further incubated for 4 h at 37 °C. The formazan crystals were formed after the lysis of cells and then dissolved using DMSO (100 µL). The absorbance of the pure LT and LT-NVoptG were evaluated at 570 nm using the microplate reader. The IC_50_ values of the samples were calculated to compare the difference between them. IC_50_ was expressed as the concentration of drug needed to kill 50% of the cells. The study was performed in triplicate.

### 2.13. Irritation Study

The chorioallantoic membrane (HET-CAM) method was used to study the irritation [39]. This method is commonly used because no animal is required to perform the study. It is a sensitive alternative to the Draize test [40]. The study was performed with negative control, positive control and prepared LT-NVoptG to compare the results. Hen eggs were taken and kept in an incubator for 10 days at 37 °C with 55 ± 2% RH. The eggs were regularly rotated after 24 h for 10 days. On the 10th day, the eggs were taken out from the chamber and then the outer eggshell was removed from the air chamber side. For clear visibility of CAM from the air chamber side, sterilized normal saline solution was added. The samples negative control, positive control and prepared LT-NVoptG were added to the CAM and the cumulative scoring was noted at different time points. The cumulative irritation score from each treated egg was compared with the standard irritant. The scoring of irritation was done as per the scale of hemorrhage, lysis and coagulation. The score was calculated between 0 (no reaction) to 3 (strong reaction). The irritation score was classified as slight irritation (≤); moderate irritation (>0.8–<1.2); irritation (≥1.2–≤2); severe irritation (≥2) [39].

### 2.14. Statistical Analysis

The data are presented in triplicate and shown as mean ± SD. Graph pad Instat was used to analyze the data (GraphPad Software Inc., La Jolla, CA, USA). Data were subjected to one-way ANOVA followed by Bonferroni multiple tests to analyze statistically significant differences between samples.

## 3. Results and Discussion

### 3.1. Optimization

**Figure 1:**
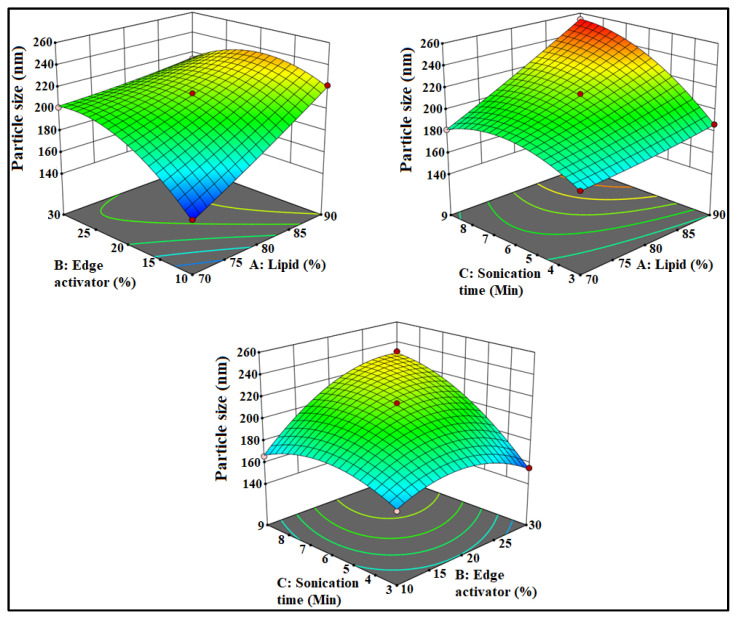
Effect of lipid (A), edge activator (B) and sonication time (C) on size.

**Figure 2:**
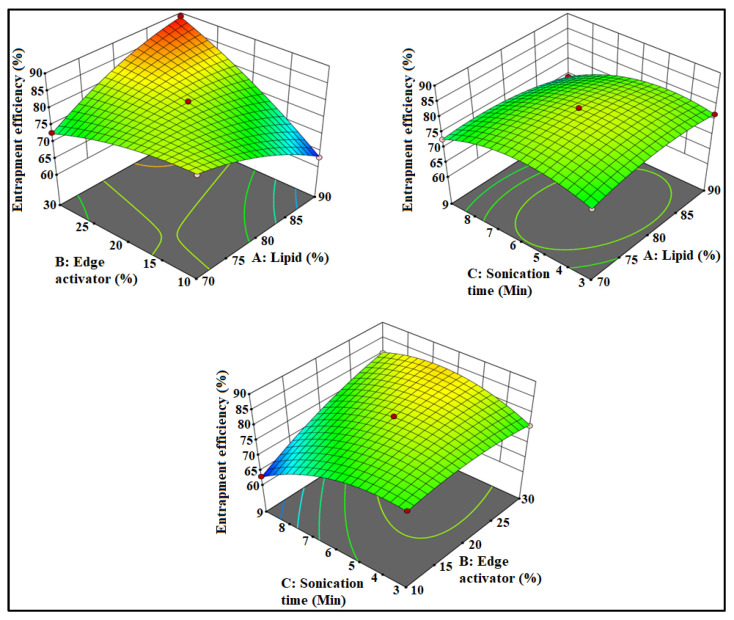
Effect of phospholipid (A), edge activator (B) and sonication time (C) on encapsulation efficiency.

**Figure 3:**
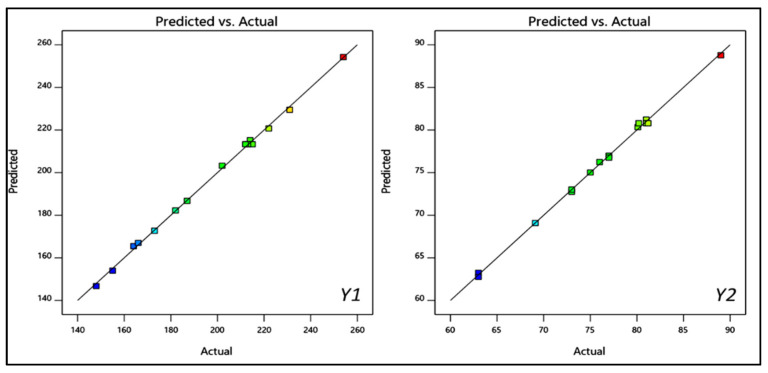
Effect of actual and predicted value of independent variables on PS (*Y*_1_) and EE (*Y*_2_).

The prepared luteolin nanovesicles (LT-NVs) were prepared by a solvent evaporation–film hydration method. BBD optimization techniques give the maximum variables at different levels along with a lower number of experimental runs [41]. The formulations were optimized using phospholipid (A), edge activator (B) and sonication time (C) as independent variables. The lower and upper levels of the independent variables were taken as phospholipid 90 G (70–90% *w*/*v*), edge activator (10–30% *w*/*v*) and sonication time (3–9 min). The design had fifteen different formulation compositions with three common compositions to check the error in the results. The used formulation variables showed a significant effect on the size (*Y*_1_) and encapsulation efficiency (*Y*_2_). The effect of formulation variables was observed by the application of the polynomial equation and response surface plot (Figure 1 and Figure 2). The independent variables showed individual as well as a combined effect on the size (*Y*_1_) and encapsulation efficiency (*Y*_2_). The different statistical parameters such as linear, cubic, quadratic and 2F models were evaluated and the best fit model was found to be quadratic. The maximum R^2^ values were found to be for the quadratic model. From the results, a closer value of predicted as well as practical value confirms that the used method composition is ideal for the prepared delivery systems. The closeness of the actual and predicted value is also shown graphically in Figure 3. Regression analysis was used to analyze the different models for dependent variables and was found to be quadratic as shown in Table 3.

### 3.2. Effect of Formulation Factors on PS

The prepared LT-NVs showed vesicle size in the range of 155.2 ± 2.9 nm (F7) to 254.6 ± 2.3 nm (F9). There was a significant (*p* ˂ 0.05) difference in the size observed between the prepared LT-NVs. The formulation F7 with the composition of phospholipid 80%, edge activator 30% and sonication time 3 min showed the lowest size. The formulation F9 showed the highest size with the composition of phospholipid 90% *w*/*v*, edge activator 20% *w*/*v* and sonication time 9 min. The used composition depicted a significant effect on the size. The polynomial equation, 3D response surface plot and contour plot (Figure 1) showed the effect of phospholipid (A), edge activator (B) and sonication time (C) on the vesicle size:

Particle size: +231.33 + 21.5 A − 12.75 B + 19.25 C − 15.5 AB + 14.5 AC + 18.5 BC + 1.58 A^2^ − 18.42 B^2^ − 15.92 C^2^.

The used factors lipid (A) and sonication time (C) showed a positive effect on the size, whereas edge activator (B) showed a negative effect. As the lipid concentration (A) increases, the vesicle size (*Y*_1_) increases. The enhancement in the size was thus due to the increase in the lipid concentration. At high concentration of lipid, the greater availability of lipid can entrap higher amounts of drug and the size increases. Therefore, the optimum concentration of lipid is important to get the optimum vesicle size. The lack of sufficient concentration of edge activator leads to low drug solubility and it also reduces the surface tension. With an increase in sonication time, the size of vesicles decreases. The phospholipid of the vesicles might rearrange to form smaller size vesicles. However, with a longer sonication time, the vesicles break into smaller size. These smaller size vesicles fold up into thermodynamically stable vesicles and the formed unstable vesicles during probe sonication may fuse together to form larger vesicles [42,43].

### 3.3. Effect of Formulation Factors on EE

The prepared LT-NVs showed the encapsulation efficiency in the range of 63.56 ± 1.9% (F10) to 89.87 ± 4.1% (F4). There was a significant (*p* ˂ 0.05) difference in the encapsulation efficiency observed due to the variation in the composition. The formulation F10 with the composition of phospholipid 80% *w*/*v*, edge activator 10% *w*/*v* and sonication time 9 min showed the minimum encapsulation and the formulation F4 showed the maximum size with composition of lipid 90%, edge activator 30% and sonication time 6 min. The effects of used composition lipid (A), edge activator (B) and sonication time (C) are depicted by the polynomial equation, 3D response plot and contour plot (Figure 2). The below given polynomial equation showed the effect on the encapsulation efficiency:

Encapsulation efficiency: +80.8 + 0.4875 A − 4.26 B − 2.48 C + 8.5 AB − 1.48 AC + 4.52 BC − 2.4 A^2^ − 1.9 B^2^ − 4.88 C^2^.

The used factor phospholipid (A) showed a positive effect on the encapsulation efficiency (*Y*_2_). With the increase in lipid concentration (A), the encapsulation efficiency of LT increases. The presence of a high concentration of lipid (A) accommodates a high concentration of lipophilic drugs, LT. The optimum concentration of lipid is important to get the optimum encapsulation efficiency because the linear increase in lipid concentration also affects the EE. The edge activator (B) showed a negative effect on the encapsulation efficiency. With the gradual increase in the edge activator concentration, the encapsulation of LT decreased. At high concentration, a greater amount of LT leaches out from the vesicles. In the case of the third factor, with the increase in sonication time (C), the encapsulation efficiency decreases. At longer sonication time, the disruption of vesicle structures as well as degradation of phospholipids takes place, resulting in higher amounts of drug leakage which leads to low EE [42].

### 3.4. Point Prediction

The selection of optimized formulation (LT-NVopt) was performed using the point prediction optimization method by further changing the independent variables. The slight change in composition also depicted changes in vesicle size and encapsulation efficiency. The optimized composition (LT-NVopt) was found to be lipid 85% *w*/*v*, edge activator 15% *w*/*v* and sonication time 4 min. This optimized composition revealed a vesicle size of 189.92 ± 3.25 nm with an encapsulation efficiency of 92.43 ± 4.12%. The software also gives predicted vesicle size of 185.11 nm with an encapsulation efficiency of 91.12%.

The used composition was also evaluated by the desirability value of the individual as well as combined independent variables. The desirability value of the prepared LT-NVs was found to be closer to 1 (0.991). The value closer to unity confirms that the used method is robust. Therefore, the LT-NVopt formulation was further converted into Carbopol gel and was characterized for different parameters.

### 3.5. Particle Size and Surface Charge

**Figure 4:**
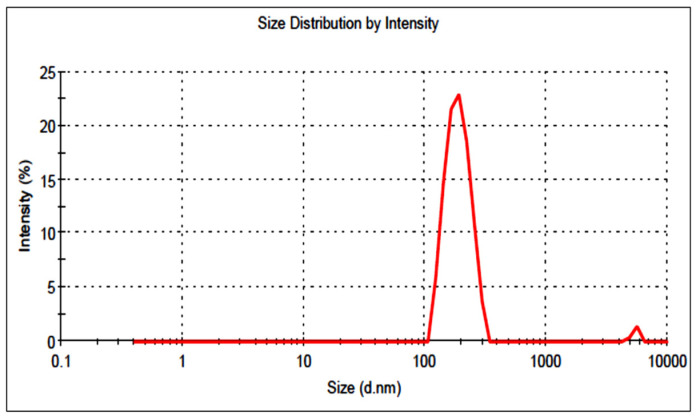
Vesicle size of optimized luteolin loaded nanovesicles (LT-NVopt).

**Figure 5:**
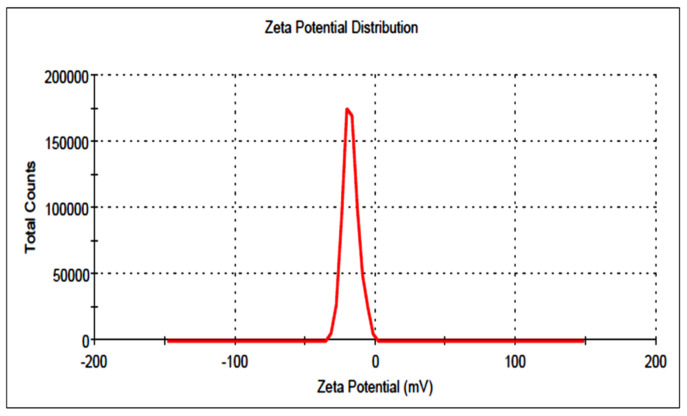
Vesicle size of optimized luteolin-loaded nanovesicles (LT-NVopt).

The prepared LT-NVs showed vesicle sizes in the range of 155.2 ± 2.9 nm (F7) to 254.6 ± 2.3 nm (F9). The optimized composition was a vesicle size of 189.92 ± 3.25 nm (Figure 4). PS less than 500 nm (100–500 nm) is ideal for cellular uptake via the endocytic pathway. In our study, prepared LT-NVopt size was found to be in the desired range of internalization by cancer cells [44,45]. The PDI and surface charge of the prepared LT-NVopt was 0.32 and −21 mV (Figure 5). Thus, less than 0.7 is considered as suitable for the delivery systems [24].

### 3.6. Formulation of Gel

The prepared LT-NVs had low viscosity and rheological properties so it was difficult to apply the skin layer. For better application, its viscosity was enhanced by the addition of Carbopol as a gelling agent. The semisolid gel system will better adhere to the skin layer. Carbopol is the most common and widely used gelling agent due to its compatibility with skin layers. The optimized concentration of Carbopol was found to be 1% *w*/*v*. At this concentration, LTNVoptG showed good rheological properties so was selected as the final gelling agent. Triethanolamine was used as a neutralizing agent to enhance the stability of the formulation [46].

### 3.7. Characterization of Gel

The prepared LT-NVopG was evaluated for different parameters such as pH, drug content, viscosity, spreadability and release study. The drug content results indicated the presence of LT in the prepared gel formulation. The prepared LT-NVoptG showed a high drug content (98.8 ± 2.12%). The high drug content is good for the adopted method as well as a delivery system. pH of the prepared LT-NVoptG was evaluated and the result was found to be closer to skin pH. The variation in pH value may lead to skin irritation. The prepared LT-NVoptG had a pH value of 6.6 ± 0.44, which is within the limit of skin formulation and does not produce any toxicity [47]. The viscosity is also one of the important parameters for the semisolid formulation. The viscosity was found to be 534 ± 1.22 cps. The particle size and PDI of a nanoformulation significantly affect the viscosity of the gel formulation. A higher particle size and PDI gives greater viscosity [48]. The prepared LT-NVoptG showed extrudability and spreadability values of 13.11 ± 1.23 g/cm^2^ and 6.2 ± 0.41 cm, respectively. The optimum range gives better application to the affected skin membrane. The high viscosity of the gel formulation gives low spreadability and extrudability values. The optimum viscosity, spreadability and extrudability results will give better adherence to the skin [49].

### 3.8. Drug Release

**Figure 6:**
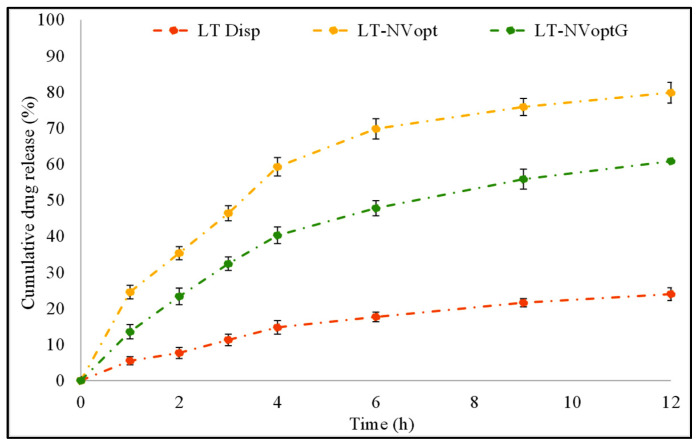
Drug release profile of pure luteolin, luteolin nanovesicles (LT-NVopt) and luteolin nanovesicles gel (LT-NVoptG). The study was performed in triplicate and data are shown as mean ± SD.

The prepared LT-NVopt, LT-NVoptG and pure LT were evaluated for drug release and the results are presented in Figure 6. The pure LT showed poor drug release (23.98 ± 1.12%) in 12 h of the study. The poor release of LT is due to the poor solubility of LT. The prepared LT-NVopt and LT-NVoptG showed a significantly (*p* < 0.001) enhanced drug release profile with a maximum drug release of 79.81 ± 3.15% and 60.81 ± 2.12%, respectively. The enhanced drug release was achieved from both the prepared formulations, which may be due to the nano size and enhanced solubility of LT in the presence of the surfactant. The nanosized vesicle has a greater surface area available to solubilize in the presence of a surfactant. There was also a significant (*p* ˂ 0.01) difference in the release observed between the LT-NVopt and LT-NVoptG. In the case of gel formulation, the drug release was found to be slower due to the presence of one extra layer of Carbopol which slowly diffuses the drug into the release media. Therefore, the LT release was found to be slower with LT-NVoptG than LT-NVopt. The slower release pattern is ideal for topical delivery because the initial fast release helps to achieve the drug concentration at the target site and later slow release helps to maintain the therapeutic concentration [50].

### 3.9. Permeation Study

**Figure 7:**
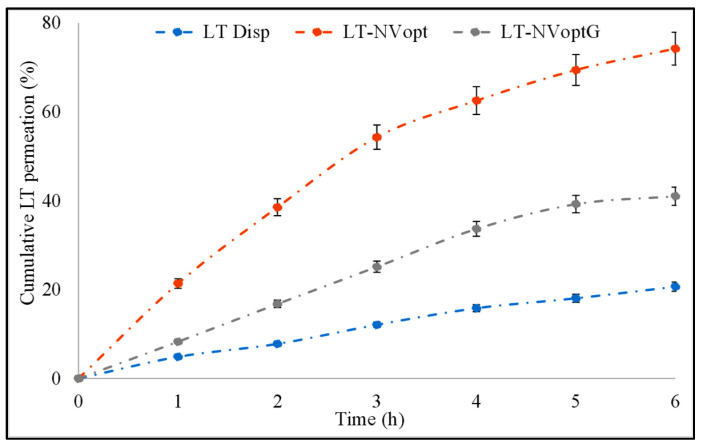
Drug permeation profile of pure luteolin, luteolin nanovesicles (LT-NVopt) and luteolin nanovesicles gel (LT-NVoptG). The study was performed in triplicate and data are shown as mean ± SD.

The comparative permeation study of LT-NVoptG, LT-NVopt and pure LT was performed to check the amount of LT which permeated across the membrane (Figure 7). The result revealed significant variation in the drug permeation profile. LT-NVopt and LT-NVoptG showed the amount of drug permeated was 231.92 ± 3.23 µg/cm^2^/h and 128.21 ± 3.56 µg/cm^2^/h in the tested 6 h study, whereas the pure LT dispersion showed the flux value of 64.59 ± 2.11 µg/cm^2^/h. There was about a 2–3.6-fold enhancement in the permeation flux achieved from LT-NVoptG and LT-NVopt. The poor permeability of pure LT is due to its poor solubility. LT-NVopt also had about 1.7-fold enhancement in the permeation compared to LT-NVoptG. LT-NVopt has enhanced permeation across the membrane due to the nanovesicle size which can easily penetrate the small pore size. LT-NVopt is prepared with sodium cholate (edge activator) which has the property to deform the membrane and penetrate across the membrane. It helps to maintain the structure of the vesicles during permeating through the tight junction of the membrane and carry the drug into the systemic circulation. [51,52]. NVoptG showed less permeation due to the slow release of LT from the gel which prolongs the drug permeation. This behavior suggests a long-lasting delivery due to the formation of a drug reservoir into the skin, which can reduce the frequency of application, thus improving patient compliance [53].

### 3.10. Antioxidant Activity

**Figure 8:**
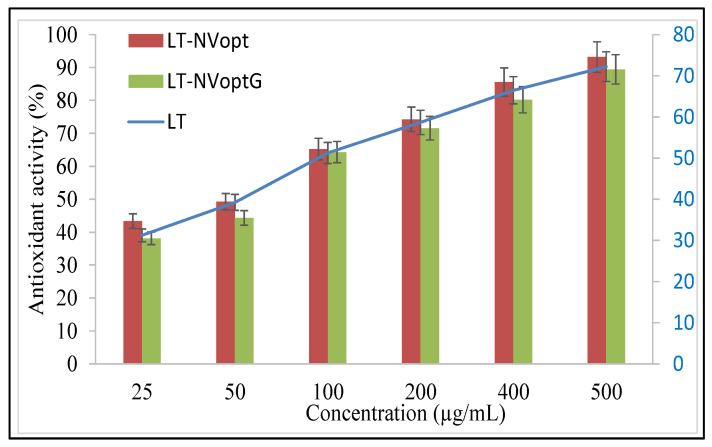
Antioxidant effects of pure luteolin (LT), luteolin nanovesicles (LT-NVopt) and luteolin nanovesicles gel (LT-NVoptG). The study was performed in triplicate and data are shown as mean ± SD.

The antioxidant potential of the prepared LT-NVopt and LT-NVoptG was evaluated using the DPPH method and the results were compared with the pure LT (Figure 8). The antioxidant potential plays an important role in the biological activity of the bioactive compound. The comparison was performed to check the effect of excipients. A significant effect was observed in the tested groups. The result was found to be concentration-dependent, so as the concentration of LT increases antioxidant potential also increases. The formulations LT-NVopt and LT-NVoptG showed significantly higher activity than the pure LT. LT-NVopt, LT-NVoptG and pure LT showed the maximum antioxidant activity of 89.18 ± 3.95%, 84.43 ± 3.11% and 70.23 ± 2.98% at 500 µg/mL, respectively. There was a significant difference in the activity observed (*p* < 0.001) at the highest concentration (500 µg/mL) in comparison to pure LT. The result was also compared between LT-NVopt and LT-NVoptG and the difference was found to be non-significant. LT-NVopt showed slightly higher activity than LT-NVoptG. The presence of gelling agents in LT-NVoptG slows the release of LT and leads to lower activity. From the results, it was observed that the DPPH-scavenging activity of LT was increased after encapsulation into lipid vesicles [36].

### 3.11. Antibacterial Activity

The antibacterial activity of prepared LT-NVoptG was evaluated and results were compared with pure LT dispersion. The pure LT-treated well showed a zone of inhibition of 13.56 ± 1.8 mm, 14.23 ± 2.1 mm and 11.76 ± 1.1 mm against *S. aureus, E. coli* and *B. subtilis*, respectively. The LT-NVoptG-treated well showed higher ZOI of 16.11 ± 2.2 mm, 15.32 ± 1.4 mm and 15.22 ± 1.9 mm, respectively. There was marked enhancement in the ZOI observed from LT-NVoptG-treated organisms. The enhancement in the activity may be due to the higher solubility of LT in the presence of the used edge activator as well as the nano size of vesicles. Due to the higher solubility of LT, it showed activity by destroying the cell wall and cell membrane and inhibiting nucleic acid synthesis [54].

### 3.12. Cell Viability

**Figure 9:**
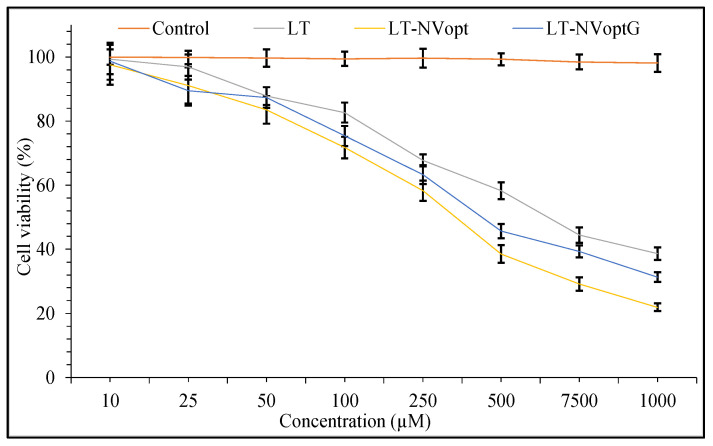
Cell viability study of pure luteolin, luteolin nanovesicles (LT-NVopt) and luteolin nanovesicles gel (LT-NVoptG). The study was performed in triplicate and data are shown as mean ± SD.

The comparative cell viability study results showed greater activity in LT-NVoptG and LT-NVopt compared to pure LT (Figure 9). LT-NVoptG and LT-NVopt revealed significantly (*p* ˂ 0.01) greater activity than pure LT. The comparison performed between LT-NVoptG and LT-NVopt also showed significant (*p* ˂ 0.05) differences among them. LT-NVoptG showed slower activity than LT-NVopt due to slower LT release from gel. LT-NVoptG- and LT-NVopt-treated cells showed higher IC_50_ values of 428.11 µM and 380 µM compared to pure LT (780.55 µM) after 24 h of treatment. The effect on the cell viability is concentration dependent. With the increase in concentration of LT, the viability % changes. The pure LT-treated cells showed the cell viability % at different concentrations of 250 µM (92.11 ± 3.2), 500 µM (69.44 ± 4.4) and 1000 µM (38.11 ± 3.8). LT-NVopt showed the cell viability % at concentration of 100 µM (85.54 ± 2.9), 250 µM (71.74 ± 4.7), 500 µM (38.54 ± 5.9) and 1000 µM (21.87 ± 2.4). LT-NVoptG-treated cells showed significant (*p* ˂ 0.001) effects at each concentration. LT-NVoptG showed the cell viability % at 100 µM (82.54 ± 2.9), 250 µM (73.65 ± 2.4), 500 µM (41.21 ± 3.6) and 1000 µM (31.11 ± 1.2). During the comparison, the difference was found to be significantly (*p* < 0.001) higher among both the groups. LT-NVoptG showed enhanced growth inhibitory effects in the treated cells at lower concentration in comparison to pure LT.

### 3.13. Irritation Study

The HET CAM method is a well-accepted method to check the irritation potential of a sample. The negative and positive control-treated sample showed a response that comes under the category of non-irritating, irritating and severely irritating. This method was applied to check the irritation potential of the prepared LT-NVoptG. The cumulative irritation score was calculated for LT-NVoptG, negative control (NaCl 0.9% *w*/*v*) and positive control (SLS, 1% *w*/*v*) to compare the results (Table 4). Visual observation was done for the lysis, hemorrhage and coagulation of blood vessels. A score between 0–0.8 is considered as non-irritant with no sign of abnormality after treatment. The negative control and prepared LT-NVoptG showed no sign of lysis, hemorrhage or coagulation. The negative control and LT-NVoptG-treated samples showed a cumulative score of 0 and 0.15 after the treatment with CAM. The positive control sample showed a cumulative score of 2.8 after treatment with CAM. The high score was shown by the positive control due to the hemorrhage and coagulation observed at different time points.

## 4. Conclusions

LT-NVs were prepared by solvent evaporation–hydration method using phospholipid, edge activator and sonication time as independent variables. The optimization of LT-NVs was performed using a Box–Behnken design with three factors at three levels. The prepared LT-NVs showed the nanometric vesicle size with high encapsulation efficiency. The optimized formulation LT-NVopt was converted to gel (LT-NVoptG) using Carbopol as a gelling agent. LT-NVoptG showed optimum viscosity, pH, spreadability, and prolonged-release profile with enhanced permeation compared to pure LT. The irritation, antibacterial and cytotoxicity results showed that the prepared LT-NVoptG was found to be non-irritating, with good antibacterial properties and lower cytotoxicity. The overall formulation design showed that the prepared LT-NVs-based gel delivery system acts as a potential delivery system in the treatment of skin diseases.

## Figures and Tables

**Table 1:** Independent variables used to optimize luteolin nanovesicles (LT-NVopt) using a Box–Behnken design.

Independent Variables	Code	Low (−1)	Medium (0)	High (+1)
Phospholipid 90 G (% *w*/*v*)	A	70	80	90
Edge activator (% *w*/*v*)	B	10	20	30
Sonication time (min)	C	3	6	9
**Dependent Variables**				
Particle size (nm)		*Y*_1_		
Encapsulation efficiency (%)		*Y*_2_		

**Table 2:** Low, medium and high levels of experimental independent variables phospholipid 90 G (A), edge activator (B), sonication time (C) with their effects on size (*Y*_1_, nm) and encapsulation efficiency (*Y*_2_, %).

Code	A (%, *w*/*v*)	B (%, *w*/*v*)	C (min)	*Y*_1_ (nm)	*Y*_2_ (%)
1	80.00	20.00	6.00	213.8 ± 1.1	81.11 ± 3.3
2	80.00	20.00	6.00	215.1 ± 1.9	80.24 ± 3.9
3	90.00	10.00	6.00	222.4 ± 4.3	73.56 ± 2.6
4	90.00	30.00	6.00	214.2 ± 1.5	89.87 ± 4.1
5	80.00	20.00	6.00	212.6 ± 2.1	81.21 ± 3.5
6	70.00	10.00	6.00	148.5 ± 3.7	81.11 ± 3.2
7	80.00	30.00	3.00	155.2 ± 2.9	76.87 ± 4.1
8	90.00	20.00	3.00	187.2 ± 1.8	77.28 ± 4.7
9	90.00	20.00	9.00	254.6 ± 2.3	69.11 ± 3.2
10	80.00	10.00	9.00	166.6 ± 4.1	63.56 ± 1.9
11	70.00	30.00	6.00	202.4 ± 3.2	73.44 ± 2.1
12	80.00	30.00	9.00	231.1 ± 1.5	80.12 ± 1.7
13	80.00	10.00	3.00	164.7 ± 1.7	77.32 ± 2.3
14	70.00	20.00	9.00	182.4 ± 2.3	73.32 ± 3.2
15	70.00	20.00	3.00	173.3 ± 3.5	75.65 ± 2.7

**Table 3:** Regression analysis summary for responses *Y*_1_ (PS) and *Y*_2_ (EE).

Model	R^2^	Adjusted R^2^	Predicted R^2^	SD
(*Y*_1_)				
Linear	0.6021	0.4924	0.2149	21.91
2F1	0.8406	0.7210	0.4411	16.25
Quadratic	0.9987	0.9963	0.9835	1.88
(*Y*_2_)				
Linear	0.2857	0.0909	−0.4251	6.68
2F1	0.8383	0.7170	0.5789	3.73
Quadratic	0.9985	0.9959	0.9876	0.45

R^2^ = Coefficient of correlation; SD = Standard deviation.

**Table 4:** HET-CAM irritation score treated with different groups.

Test Sample	Egg	Time (min)				Overall Score
		0	0.5	2	5	
LT-NVopG	Egg 1	0	0	0	0	0.15
	Egg 2	0	0	0	0	
	Egg 3	0	0	0.2	0.4	
	Egg 4	0	0	0	0	
	Mean score	0	0	0.05	0.1	
SLS, 1% *w*/*v* (Positive control)	Egg 1	0	0.8	0.8	2	
	Egg 2	0	0.8	1.2	2	2.8
	Egg 3	0	0.8	1.2	1	
	Egg 4	0	0	0.8	0	
	Mean score	0	0.6	1	1.2	
NaCl 0.9% *w*/*v* (Negative control)	Egg 1	0	0	0	0	
	Egg 2	0	0	0	0	0
	Egg 3	0	0	0	0	
	Egg 4	0	0	0	0	
	Mean score	0	0	0	0	
